# qPCR detection of viable *Bacillus cereus* group cells in cosmetic products

**DOI:** 10.1038/s41598-023-31128-3

**Published:** 2023-03-18

**Authors:** Nadine Yossa, Sonny Huang, Travis Canida, Rachel Binet, Dumitru Macarisin, Rebecca Bell, Sandra Tallent, Eric Brown, Thomas Hammack

**Affiliations:** 1 FDA, Office of Regulatory Science, College Park, MD USA; 2 Oak Ridge Institute for Science and Education, Oak Ridge, TN 37831 USA; 3 FDA, Office of Analytics and Outreach, College Park, MD 20740 USA

**Keywords:** Applied microbiology, Environmental microbiology

## Abstract

Reference methods for microbiological safety assessments of cosmetics rely on culture methods that reveal colonies of live microorganisms on growth media. Rapid molecular technologies, such as qPCR, detects the presence of target DNA in samples from dead and viable cells. DNA intercalating dyes, such as propidium monoazide (PMAxx), are capable of restricting PCR amplification to viable microbial cells. Here we developed singleplex and multiplex real time (qPCR) assays for the detection of *Bacillus cereus* (*B. cereus*) using 16S rRNA and phosphatidylcholine-specific phospholipase C (PLC) gene specific sequences coupled with PMAxx. The limit of detection was determined to be ~ 1 log CFU/ml for 16S rRNA and 3 log CFU/ml for PLC detection in pure culture using an eye shadow isolate, *B. cereus* 3A. We assessed the inclusivity and exclusivity of our qPCR assays using 212 strains, including 143 members of *B. cereus*, 38 non- *B. cereus*. and 31 non-*Bacillus* species; inclusivity was 100% for the 16S rRNA and 97.9% for the PLC targets; the exclusivity was 100% for 16S rRNA and 98.6% for PLC targets. These qPCR assays were then used to assess samples of commercial cosmetics: one set of liquid face toners (N = 3), artificially contaminated with *B. cereus* 3A, and one set of powdered cosmetics (N = 8), previously determined to be contaminated with *B. cereus*. For some samples, test portions were analyzed by qPCR in parallel, with and without PMAxx treatment. All test portions were simultaneously streaked on BACARA plates to confirm viable cells of *B. cereus*, according to the culture method. We found no difference in sensitivity between the singleplex and the multiplex qPCR assays (*P* > 0.05). Inoculated samples that did not recover *B. cereus* on plates still showed amplification of the DNA targets. However, that amplification was significantly delayed in PMAxx –treated samples (*P* < 0.0001) with C_T_ value differences of 7.82 for 16S rRNA and 7.22 for PLC. Likewise, amplification delay was significant (*P* < 0.0001) with inoculated samples that recovered *B. cereus* on plates with C_T_ value differences of 2.96 and 2.36 for 16S rRNA and PLC, respectively, demonstrating the presence of dead cells in the samples. All our qPCR results correlated with detection on BACARA plates (kappa, k = 0.99), independently of the presence of PMAxx in the PCR assays. Nevertheless, the amplification threshold with PMAxx dyes was significantly higher than the non-PMAxx dyes. Our findings confirm qPCR can be used for more rapid detection of microorganisms in cosmetics, including *B. cereus*, and selective detection of viable cells can be improved using PMAxx dyes.

## Introduction

Although cosmetics do not need to be sterile, to protect the safety of people who use cosmetics, bacterial contamination above 1000 colony forming units (CFU/g or ml) is grounds for regulatory action in the United States (USA) and removal of contaminated products from the market; for products used around the eye area, this limit is 100 CFU/g or ml^1^. As these grounds for action are culture-based, testing to assess cosmetics for contamination has also remained culture-based. In the United States, detection of *B. cereus* in cosmetics is typically performed using the culture-based methods described in the Bacteriological Analytical Manual (BAM) of the U.S. Food and Drug Administration^2^ (Tallent, Knollhoff et al. 2021). The reference techniques for cosmetics, described in Chapter 23 of the BAM, use selective and chromogenic media, biochemical confirmation, and phenotypic characterizations (e.g., cell and spore morphology, rhizoid growth, and the presence of specific proteins) for bacterial identification, which can take up to 7 days, including steps to pre-enrich cultures to support detection of low-level microorganisms^1^. Although molecular methods are faster, the sensitivity of molecular methods means these methods may detect the presence of bacterial DNA whether or not the organisms from which that DNA comes are still alive^3^. Specifically, preservatives in cosmetics products are expected to kill or damage microbial cells that may be present during manufacture or introduced during use by the consumer. The process of sample enrichment during testing of potentially contaminated products revives and amplifies damaged cells. DNA of these cells, along with that from any dead microorganisms, can be amplified by qPCR. This can result in overestimating the amount of live microorganisms’ present^4^. This problem potentially prevents the accurate comparison to regulatory standards based on CFUs.

Therefore, if rapid methods are to become accepted for cosmetics testing, some way must be found to preferentially identify live bacteria in cosmetics.

One method for preferentially improving the detection of viable cells uses intercalating nucleic acid dyes that penetrate only into dead and compromised membrane cells. Once inside the cell, these dyes intercalate covalently into the DNA after photoactivation with light and thereby interfere with the amplification of that DNA. These dyes include ethidium monoazide^5^, propidium monoazide^6^, and propidium monoazide (PMAxx, Botium 2016), which can be used to pretreat samples prior to performing qPCR. A wide variety of microorganisms have been assessed using these dyes, including: viruses^7^, fungi^8^, protozoa^9^, and foodborne pathogens such as *Escherichia coli* O157:H7^10^, *Listeria monocytogenes*^11^ and *Salmonella*^12^; *Campylobacter jejuni*^13^, *Staphylococcus aureus*^14^ and *B. cereus*^15^.

About *B. cereus.*

The *B. cereus* group species complex, also known as *B. cereus* sensu lato (s.l.), is a subgroup of closely related species belonging to the genus *Bacillus*. Group members are Gram-positive, spore-forming, and widely distributed throughout the environment^16^. This *B. cereus* group comprises at least twelve closely related species: *B. anthracis*, *B. cereus*, *B. thuringiensis*, *B. mycoides*, *B. pseudomycoides*, *B. weihenstephanensis*, *B. cytotoxicus*, *B. wiedmanni*, *B. toyonensis*, and the recently identified *B. paranthracis, B. pacificus, B. tropicus, B. albus, B. mobilis, B. Luti, B. proteolyticus, B. nitratireducens, B. paramycoides, B. gaemokensis, B. manliponensis, B. bingmayongensis*, and *B. fungorum*^17^. The best-known members of this group are *B. anthracis*, *B. cereus* and *B. thuringiensis*, each of which can have a significant impact on human health, agriculture, and the food industry^18^. Rapid detection of *B. cereus* and its close relatives is important because *B. cereus* group members can produce a variety of hemolysins, phospholipase C (PLC), emetic toxins, enterotoxins, metalloproteases, collagenases, and beta-lactamases^19,20^ that cause gastrointestinal diseases and localized infections, such as wound and eye infections^21^. While *B. cereus* has not been a major concern for cosmetics, its presence is still undesirable, and developing rapid detection methods applicable to cosmetic products would be advantageous.

Detection methods using PCR and qPCR have already been established for identifying *B. cereus* in foods, for example: targeting the 16S rRNA gene in naturally contaminated food gelatin^22^, infant food^14,23^, and in food contact surfaces, such as cardboard and paper^24^. The phosphatidylcholine-specific phospholipase C (PLC) gene has been detected in artificially contaminated liquid eggs and reconstituted infant formula^20^. PLC gene was chosen in this study because of its prevalence in *B. cereus*^25^ and contribution in the pathogenicity of eye infection^26^.

To our knowledge, qPCR targeting the 16S rRNA and PLC genes have not yet been used formally to detect *B. cereus* in cosmetics. Therefore, here we present the development and use of two qPCR assays (simgleplex and multiplex) for the detection of *B. cereus* in powdered and liquid cosmetics, which may help bridge the gap between culture-based and rapid molecular methods of detection.

To ensure our qPCR is fit for purpose to assess bacterial contamination in cosmetics, we first established the limit of detection (LOD) using a known *B. cereus* isolate from eye shadow cosmetic product (“3A”), then we performed inclusivity tests using a panel of 143 *B. cereus* group members to determine that our method can detect a broad range of *B. cereus* strains. Then we performed exclusivity tests using 38 non-*B. cereus* and 31 non-*Bacillus* to establish that the method did not amplify non-target strains or species.

Finally, we assessed how well these qPCR assays (with or without PMAxx, and in both singleplex and multiplex formats) could detect the 16S rRNA and PLC genes of *B. cereus* in naturally or artificially contaminated cosmetics (powder and liquid types) and we directly compared this performance with outcomes using the standard culture method described in BAM Chapter 23^1^.

## Materials and methods

### Bacterial strains and preparation

The 212 bacterial strains used in this study were obtained from cosmetics, food, environmental sources, as well as from the American Type Culture Collection (Table 1).

**Table 1:** Bacterial strains used to verify inclusivity and exclusivity.

Species Category	Number
*B. cereus* group members	143
Non-*B. cereus*	38
Non-*Bacillus* species	31

All 212 strains used in this study were maintained at − 80ºC in broth supplemented with 20% glycerol. Each strain was aseptically streaked onto Tryptic soy agar (TSA) (Difco™, Franklin Lakes, NJ) and incubated for 24 h at 30ºC, from which an isolated colony was sub-cultured in Nutrient Broth (NB, pH 7.2) (Difco™), then incubated at 30ºC for 24 h.

Inclusivity testing was performed using 143 members of the *B. cereus* group. One of these strains, *B. cereus* “3A”, previously obtained from eye shadow (Yossa and Jo Huang, personal communication), was used to establish the limit of detection (LOD) for our assays. Exclusivity testing was performed using a panel of 69 strains: 38 non-cereus strains of *Bacillus*, and 31 strains of non-*Bacillus* bacteria.

### Cosmetic products

There were two types of cosmetics used in this study: liquid and powder. To assess how well our assays could detect the presence of *B. cereus* 3A in artificially contaminated samples of liquid-type cosmetics, we purchased facial toner products [N = 3], all of which were labeled “alcohol free”. These facial toners were primarily composed of water and plant-derived compounds and preserved with phenoxyethanol, an antibacterial agent.

To assess the performance of our assays on powder-type cosmetics, we selected 8 cosmetic products: Green Clay (GC), Pink Clay (C1–4), Rice Powder (RP), and Tattoo Powder (O1–2), which had been purchased from a retail establishment online. The two clay products contained no preservatives, the rice powder product contained phenoxyethanol, and no information was available about any preservative in the tattoo product. Prior research had determined 6 powder-based products to be contaminated with *B. cereus* (Yossa and Jo Huang, personal communication); therefore, we classified these as “naturally-contaminated” and did not add any additional bacteria to these powders.

### DNA extractions

#### Inclusivity, exclusivity, limits of detection

We isolated DNA from overnight cultures of each pure bacterial strain using the MagMAX™ Express 96 Magnetic Particle Processor (ThermoFisher) with PrepSEQ Nucleic Acid Extraction Kit for Food and Environment (ThermoFisher, P/N 4,428,176) using protocol PrepSeq_ResDNA_20011 (Life Technologies, Carlsbad, CA). Total DNA extracted from these pure cultures were used to evaluate the inclusivity (n = 143) and exclusivity (n = 69) of the qPCR assays.

To determine the limit of detection of our qPCR assays, we used DNA extracted from the *B. cereus* 3A strain, which was grown overnight in NB and adjusted to a density of 0.5 ± 0.05 MacFarland (McF; ~ 6.7 log CFU/ml).

#### DNA extractions from cosmetics

Two different extraction procedures were used to perform our qPCR assays on the sets of contaminated cosmetics, due to differences in the matrices. The MagMAX™ and PrepSeq kits were only appropriate for liquid cosmetics. Effective DNA extraction from the powder products required using the DNeasy® PowerPlant® Pro kit (Qiagen, Catalog Number 69204) instead, because powder products become thick and dense in the presence of the proteinase/proteinase DNA extraction buffer used in the MagMAX and PrepSeq kits^27^.

#### Primers/probes

Table 2 shows the primers we used and the associated publications documenting their first use. These were purchased from Life Technologies (ThermoFisher).

**Table 2:** PCR Primers used for the multiplex qPCR assays.

Target genes	Sequences (5’–3’)	References
16S rRNA	F: GCG GCG TGC CTA ATA CAT GC	^28^
	R: CTC AGG TCG GCT ACG CAT CG	
	Probe: FAM-TCG AGC GAA TGG ATT AAG AGC TTG C-BHQ1	^22^
PLC	F : GGA TTC ATG GAG CGG CAG TA	^20^
	R: GCT TAC CTG TCA TTG GTG TAA CTT CA	
	Probe: JUN-CGA AAG ATT ACT C -QSY	Designed and modified by Life Technologies
IPC	TaqMan Exogenous Internal Positive Control (P/N 4,308,323)	Designed and modified by Life Technologies

*16S rRNA* 16S ribosomal DNA, *PLC* phosphatidylcholine-specific Phospholipase C, *IPC* Internal positive control.

The 16S rRNA sequences (P/N 4,331,348, Life Technologies) used here had previously been determined by De Clerck, et al.; their team targeted the 5’ hypervariable fragment, which can be amplified using a universal forward and a *B. cereus*-specific reverse primer^28^. Our TaqMan probe was labeled with 6 -carboxyfluorescein (FAM) reporter dye at the 5’ end and labeled at the 3’-end with a Black Hole quencher, BHQ1, to reduce background fluorescence^29^. We ordered the PLC primer probe (CCU001SNR, Life Technologies) labeled with a JUN reporter dye at the 5’-end and a non-fluorescent quencher at the 3’-end, to facilitate multiplex qPCR using 4 dyes. QSY PLC- JUN probe is a custom probe, compatible with the TaqMan Multiplex Master Mix (P/N 446,188; Applied Biosystems, Life Technologies) and ordered through Life Technologies.

The TaqMan Internal Positive Control (IPC) from Life Technologies (P/N 4,308,323) was included to monitor the PCR progress and ensure that a negative result is not caused by failed PCR in the sample^30^ and used with the TaqMan Multiplex Master Mix to amplify both the single target and the multiple target reactions.

#### qPCR reactions of non-treated and PMAxx -treated samples

PMAxx -PCR is an innovative technology that allows differentiation between live and dead microorganisms, based on the loss of cell membrane integrity in dead cells^31^. This system uses a DNA-intercalating dye, PMAxx, that disrupts DNA transcription only in dead cells, as their damaged cell membranes permit entry of the dye. After photoactivation with a defined wavelength, PMAxx intercalates and binds covalently to DNA. Subsequent amplification of that modified DNA is inhibited, thereby reducing the amplification signal from dead/damaged cells in comparison to that from live cells.

For each qPCR reaction, a 20 μl volume consisting of 10 μl Multiplex Master Mix, 2 μl 10X Exo IPC Mix, 0.4 μl 50X EXO IPC DNA, 1 μl of primer assay, 2 μl of sample/template DNA, and 4.6 μl of sterile deionized water was placed into a 96 well fast plate. Both primers for the multiplex PCR were combined to reach a final concentration representing 5% of the total reaction volume. These qPCR runs were performed on a 7500 Fast qPCR System (Applied Biosystems) under the following conditions: 2 min at 50 °C, then 10 min at 95 °C, followed by 40 cycles of 15 s at 95 °C and 1 min at 60 °C.

#### *B. cereus* 3A testing in artificially contaminated liquid products

Here we used 3 different cosmetic toner products, which we artificially contaminated with *B. cereus* 3A. For each trial (N = 3), 3 sterile Wheaton bottles (125 ml) were filled with 30 ml of facial toner and were repeatedly spiked with 300 μl of microbial suspension, containing either a High (~ 4 or 3 log CFU/ml) or Low (~ 3 or 2 log CFU/ml) level of *B. cereus* 3A vegetative cells (optical density of 0.5 ± 0.05 McF), over three consecutive days, and the 3rd bottle of sample remained un-spiked for the negative controls. These repeated inoculations were necessary to enable recovery of *B. cereus* from products containing preservatives^32^. To mimic a product contamination event more closely, these inoculated toner samples, along with the un-spiked negative control, were aged for 14 days at room temperature. After 14 days of aging, the un-inoculated and inoculated samples from the High- and Low-level inoculation were gently separately mixed, then 1 ml aliquots were diluted with 9 ml MLB (Difco).

**Figure 1:**
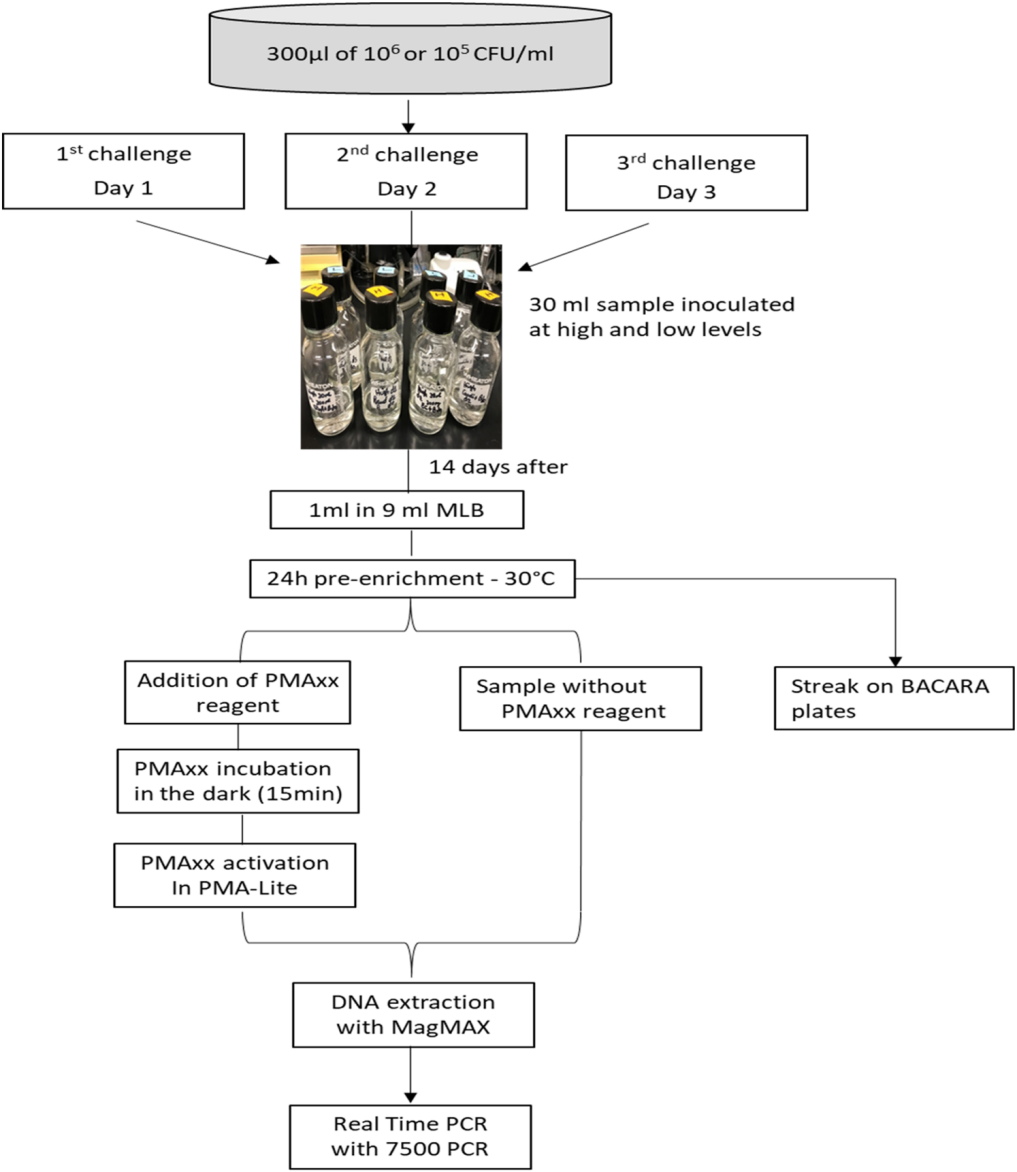
Overview of the workflow of the PMAxx-treated vs non-treated samples. *MLB* modified letheen broth, *PMAxx* modified propidium monoazide.

Our first goal was to achieve fractional results, which in this case would consist of 5 replicates at the high level of inoculation all yielding positive results, while 20 replicates at the low level of inoculation would yield only 50 ± 25% positive results, and 5 replicates of un-inoculated sample all yielding negative results^33^. Based on this, 5 replicates of toner from the High level, 20 replicates from the Low level, and 5 replicates from the uninoculated samples (negative controls) were pre-enriched for 24 h at 30 °C in MLB, streaked onto BACARA plates and processed simultaneously for molecular analysis. Two equal sets of test portions were taken at the same time in all the replicates and placed into vials, and the first set of test portions proceeded to genomic DNAs extraction without any treatment. From the second set, 1 ml of each test portion was supplemented with 25 µM PMAxx, mixed through tap-spin, and allowed to incubate at room temperature for 10 min in the dark before exposure on the PMA-Lite (Biotium) for 15 min to crosslink free DNA^34^. Immediately following PMAxx treatment, genomic DNAs were extracted, and all genomics (non-treated and treated) were analyzed independently in duplicate using singleplex and multiplex qPCR assays. Figure 1 underlines an overview of the workflow of this portion of our study.

#### Microscopic observation of live/dead *B. cereus* cells aged for 14 days in toner-samples using BacLight kit

To provide a microscopic perspective on the ratio of live/dead cells in the inoculated toner samples, 1 ml from the 14-day aged samples inoculated with *B. cereus* at the high level was centrifuged at 10,000 × g for 10 min. The supernatant was removed, and the pellet was resuspended in 1 ml of 0.85% sodium chloride (NaCl) and kept at room temperature for 1 h, mixing every 15 min. After centrifugation at 10 000 × g for 10 min, the cells were resuspended in NaCl and stained with the Live/Dead™ Bacterial Viability kit (Cat: L7007, Invitrogen by ThermoFisher Scientific) according to manufacturer’s protocols^35^. Stained cells were viewed under a Zeiss 880 Laser Scanning Microscope (LSM, Carl Zeiss Microscopy, LLC, White Plains, NY). The cells were observed using a Zeiss Axio Observer inverted microscope with × 63 1.4 NA oil immersion plan apochromatic objective. Differential interference contrast (DIC) and confocal fluorescence images were acquired simultaneously. A photomultiplier tube captured the light emitted from a 488-nm argon laser with a 3.7-m pin hole passing through an MBS 488 filter with limits set between 472 and 562 nm for detection of SITO 9 stains for green fluorescence of live cells and between 597 and 669 nm for detection of propidium iodide stains for red fluorescence of bacterial cells with damaged membrane. Zeiss Zen Black software was used to obtain the images with 1024 × 1024-pixel resolution.

#### *B. cereus* testing in powder-type cosmetic products

Each of the 8 powder-types cosmetic products (GC, C-1, C-2, C-3, C-4, O-1, O-2 and RP) was aseptically homogenized, and 1 g was added to 1 ml of Tween 80, before mixing with 8 ml of MLB broth in presence of 10 sterile glass beads to ameliorate homogenization. These samples were diluted, and the 10^−1^–10^−2^ dilutions were spread on MLA and BACARA plates, before enrichment at 30 °C for 24 h. Spread plating was used instead of spiral plating, to prevent powder particles from clogging the stylus of the spiral plater. All these enriched samples were streaked onto BACARA plates to screen for *B. cereus* presence, according to BAM Chapter 23^1^ and processed simultaneously to extract bacterial DNA for *B. cereus* qPCR analysis, in duplicate. This will permit cross-method comparisons.

#### Statistical analysis

Data were analyzed using a linear mixed effects model and *p*-values were adjusted to account for multiple comparisons. Cutoff points were established using a logistic regression model, such that the cutoff Ct value gives a > 50% chance of a sample being negative. Inclusivity and exclusivity cutoff values were established using the upper bound of the 95% confidence interval of the 95th quantile estimate^36^. The linear mixed effects model was fit using the nlme package version 3.1–145^37^. Quantile regression was done using the quantreg package version 5.55. All other analyses were done using R version 3.6.0^38^.

### Ethics declarations

This research does not involve human, animal, seed or plant samples.

## Results

### Assessing sensitivity of the qPCR using *B. cereus* 3A

**Figure 2:**
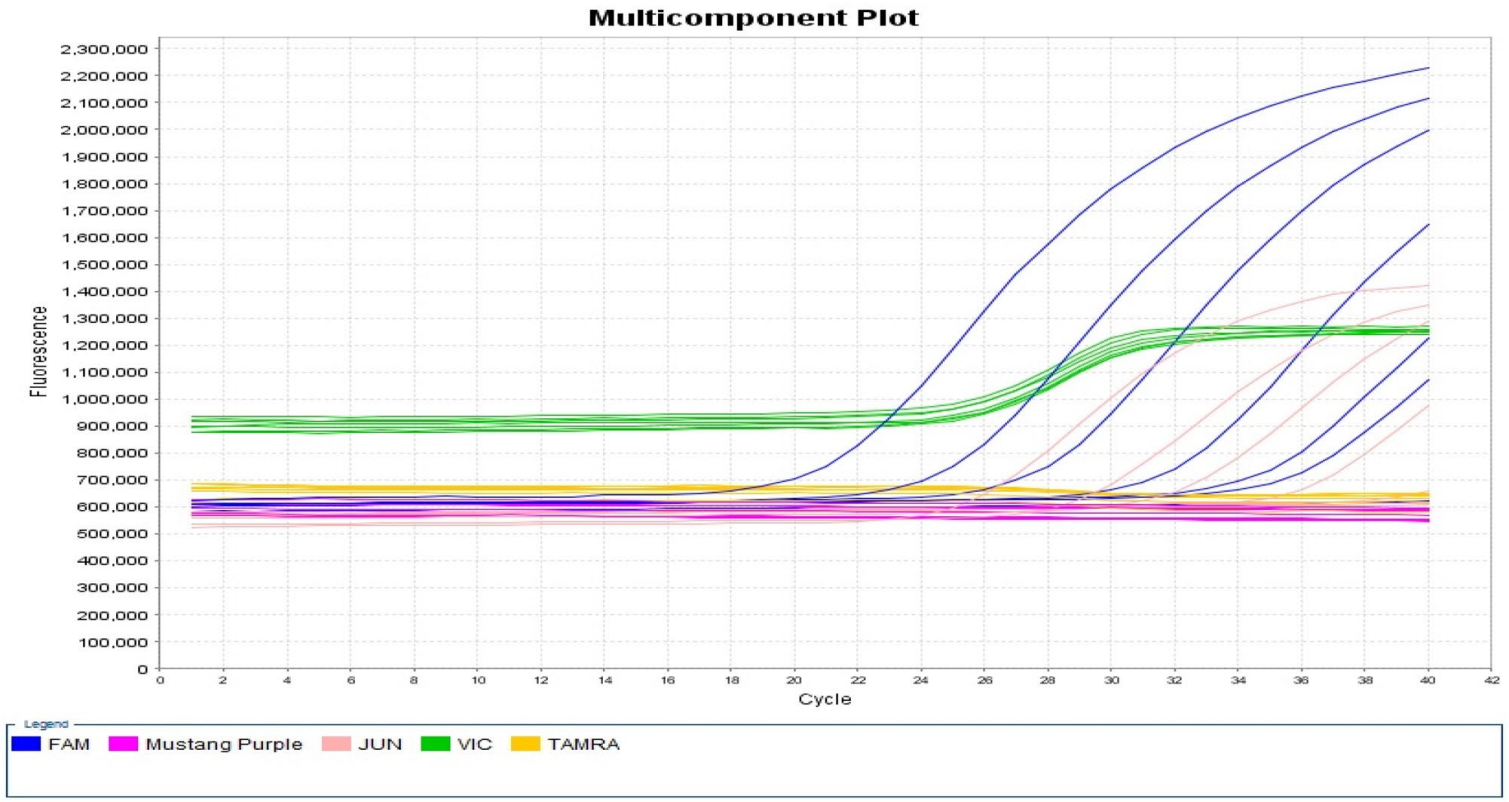
Tenfold serial dilution multiplex amplification of 16S rRNA and PLC of B. cereus 3A The Blue line for the reporter FAM represents 16S rRNA from d0 to d6 corresponding C_T_ values of 21.28, 24.95, 27.99, 31.99, 35.09, 36.23 and undetermined. The Fuchsia line tracks the passive reference dye, Mustang Purple providing an internal reference for normalizing the reporter dye signal during analysis. The Pink line for the reporter JUN represents PLC from d0 to d4 with C_T_ values of 26.29, 29.90, 32.90, 36.98; dilutions 5 and 6 were undetermined. The Green line for the reporter VIC represents the Internal Positive Control amplification used to ensure the quality of the reagents and the instrument. The Yellow line represents the quencher TAMRA. *PLC* phosphatidylcholine-specific phospholipase C.

The limits of detection (LOD) for our qPCR assays, in singleplex and multiplex formats, were determined in two independent runs with tenfold serial dilutions (d0–d6) from purified genomic DNA of *B. cereus* 3A at, a density of 0.5 McF equivalents, to ~ 6 log CFU/ml. As shown in Table 3, the LOD for the singleplex and multiplex assays were found to be ~ 1 log CFU/ml (C_T_: 36.61 and 36.23) for 16S rRNA detection and 3 log CFU/ml (C_T_:36.32and 37.72) for PLC detection. A multiplex multicomponent of the tenfold serial dilution (n = 1) is represented in Fig. 2.

**Table 3:** Determination of the detection limit for *B. cereus* 3A* using qPCR C_T_ values.

Estimated cells numbers per ml (serial dilution)	Singleplex		Multiplex	
	16S rRNA	PLC	16S rRNA	PLC
1 × 10^6^ (d0)	21.01 ± 0.92	26.50 ± 1.02	21.88 ± 0.85	26.81 ± 0.73
1 × 10^5^ (d1)	24.68 ± 1.03	29.75 ± 0.93	25.56 ± 0.86	30.37 ± 0.67
1 × 10^4^ (d2)	28.43 ± 1.42	33.39 ± 1.39	28.99 ± 1.42	33.77 ± 1.23
1 × 10^3^ (d3)	32.03 ± 1.49	36.32 ± na	32.84 ± 1.19	37.72 ± 1.05
106.75 (d4)	35.33 ± 2.03	39.70 ± na	36.31 ± 1.73	Und
17 (d5)	36.61 ± na	Und	36.23 ± na	Und
0.75 (d6)	Und	Und	Und	Und

*Tenfold serially diluted (d0–d6) *B. cereus* 3A. These data are averaged across 2 separate runs, *na* Not averaged: only one signal was read, *Und*. Undetermined, no signal was observed; *16S rRNA* 16S rRNA, *PLC* phosphatidylcholine-specific phospholipase C.

Figure 2 shows the multiplex amplification of 16S rRNA and PLC for the tenfold dilution series of *B. cereus* 3A.

### Inclusivity and exclusivity of the qPCR assays

Inclusivity and exclusivity results are summarized in Table found in supplemental materials, along with the origins for each strain. Inclusivity was determined using 24 h actively grown pure cultures of *B. cereus* vegetative strains and closely related species belonging to the *B. cereus* group (N = 143) (Table in supplemental material). Among these 143 strains, inclusivity for the 16S rRNA was 100% for with a C_T_ cutoff value of 23.8–23.9 and for PLC it was 97.9% (140/143) with a C_T_ cutoff value of 26.6–28.2 for both the singleplex and the multiplex qPCR. All the strains belonging to *B. cereus* group were confirmed by growth on BACARA plates, where these exhibited the typical morphology of orange colonies surrounded by a white halo.

Exclusivity was determined using 69 strains: 38 non-*B. cereus* and 31 non-*Bacillus* species. Exclusivity for 16S rRNA was 100% for both non-*cereus* group and non-*Bacillus* species. However, the exclusivity tests for PLC showed 98.6% (68/69) for non-*cereus* group (37/38) and 100% for non-*Bacillus* species (31/31**).** Detection of *B. cereus* 3A in artificially contaminated liquid-type cosmetics, using culture and molecular methods, in presence or absence of PMAxx -dye.

After the pre-enrichment step, low-level inoculation (~ 2 Log CFU/ml) of *B. cereus* 3A yielded fractional positive results (75% of the test portions were positive) while 100% samples from the high-level inoculation (~ 3 Log CFU/ml) were positive.

**Figure 3:**
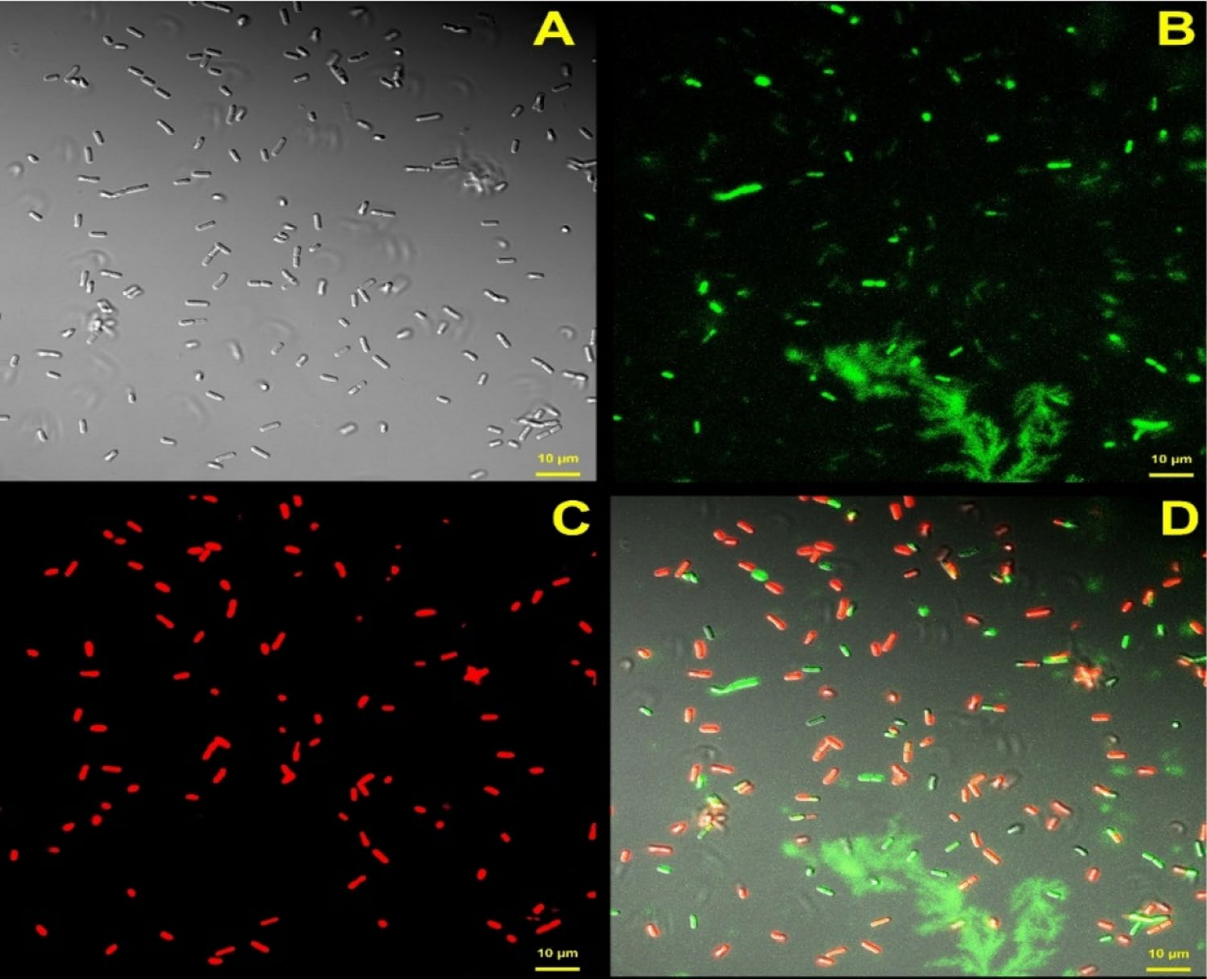
Laser Scanning Confocal microscopy imaging of High-level inoculated *B. cereus* cells in the liquid-type cosmetic aged for 14 days and then stained with Live/Dead dye. Live cells are green and dead/injured cells fluoresce red. (**A**): DIC, Differential interference contrast; (**B**): green channel; (**C**): red channel; (**D**): all channels.

Observations of the bacterial population in samples inoculated at the High level, aged for 14 days, then stained with the Live/Dead™ Bacterial Viability kit, showed a mixture of live and dead cells, which had likely been killed by the preservatives in the cosmetic (Fig. 3). Cells were not detectable in the Low level of inoculation. The negative control, consisting of uninoculated samples, showed neither growth nor DNA amplifications. qPCR C_T_ values of those samples were undetermined.

The C_T_ results for16S rRNA and PLC obtained from non-treated and PMAxx -treated samples in singleplex were not significantly (*P* > 0.05) different from the C_T_ results in multiplex qPCR. In addition, those results agreed with the results on BACARA plates (Cohen’s Kappa κ = 0.99; Tables 4, 5).

**Table 4:** Detection of viable cells of *B. cereus* 3A in in artificially contaminated liquid-type samples using singleplex PMAxx -qPCR and BACARA plates.

*B. cereus* 3A	Treatment	16S rRNA C_T_	16S ΔC_T_	PLC C_T_	PLC ΔC_T_	BACARA
Vegetative cells	Non-treated	25.79 ± 0.43	7.82*	31.81 ± 0.34	7.22*	no growth (dead cells)
	PMAxx -treated	33.61 ± 0.53		39.03 ± 0.53		no growth (dead cells)
	Non-treated	13.97 ± 0.25	2.96*	19.99 ± 0.25	2.36*	Growth (live cells)
	PMAxx -treated	16.93 ± 0.27		22.35 ± 0.26		Growth (live cells)

*indicates a statistically significant difference (*P* < 0.0001), Δ*C*_*T*_ indicates the differences between PMAxx -treated and non-treated C_T_ values. N = 3 trials.

**Table 5:** Detection of viable cells of *B. cereus* 3A in in artificially contaminated liquid-type samples using multiplex PMAxx -qPCR and BACARA plates.

*B. cereus* 3A	Treatment	16S rRNA	16S ΔC_T_	PLC	PLC ΔC_T_	BACARA
Vegetative cells	Non-treated	26.80 ± 0.34	7.79*	31.32 ± 0.34	7.22*	no growth (dead cells)
	PMAxx -treated	34.59 ± 0.53		38.54 ± 0.53		no growth (dead cells)
	Non-treated	14.95 ± 0.25	2.96*	19.50 ± 0.25	2.36*	Growth (live cells)
	PMAxx -treated	17.91 ± 0.26		21.86 ± 0.26		Growth (live cells)

*indicates a statistically significant difference (*P* < 0.0001), Δ*C*_*T*_ indicates the differences between PMAxx -treated and non-treated C_T_ values. N: 3 trials.

As expected, the C_T_ values for non-treated samples were significantly different (*P* < 0.05) from those for the PMAxx -treated samples. Non-PMAxx treated test portions from samples that yielded colonies when plated on BACARA (positive samples according to the culture method), yielded mean 16S rRNA 13.97 ± 0.25 in singleplex and 14.95 ± 0.25 in multiplex, and C_T_ values for PLC of 19.99 ± 0.25 in singleplex and 19.50 ± 0.25 in multiplex. Table 4 shows the delta differences across the two conditions, i.e., the mean C_T_ value of the PMAxx -treated samples minus the mean C_T_ value of the non-treated samples. The untreated samples show C_T_ values delta C_T_ value differences of 2.96 and 2.36 cycles for 16S rRNA and PLC, respectively, which are significantly lower (*P* < 0.0001) than the C_T_ values of the culture-positive samples that were treated with PMAxx (Table 4). Likewise, the mean C_T_ values obtained for the non-treated test portions which did not yield colonies on BACARA plates (culture-negative samples) were significantly lower than the mean C_T_ values of the PMAxx -treated samples with a delta difference of 7.82 and 7.22 for 16S RNA and PLC, respectively, for singleplex and multiplex (Table 5).

The C_T-_shifts of the positive samples (live cells) treated with PMAxx ranged between 2–3 C_T_ values: this is due to the presence of a portion of dead cells. On the other hand, the C_T-_shifts of the negative samples (dead cells) were more prominent ranging between 7 and 8 C_T_ values making it clear to distinguish between dead and live cells. PMAxx treatment does preferentially improve the detection of live cells.

### Results of *B. cereus* testing in powdered cosmetics using culture-based and molecular methods

**Figure 4:**
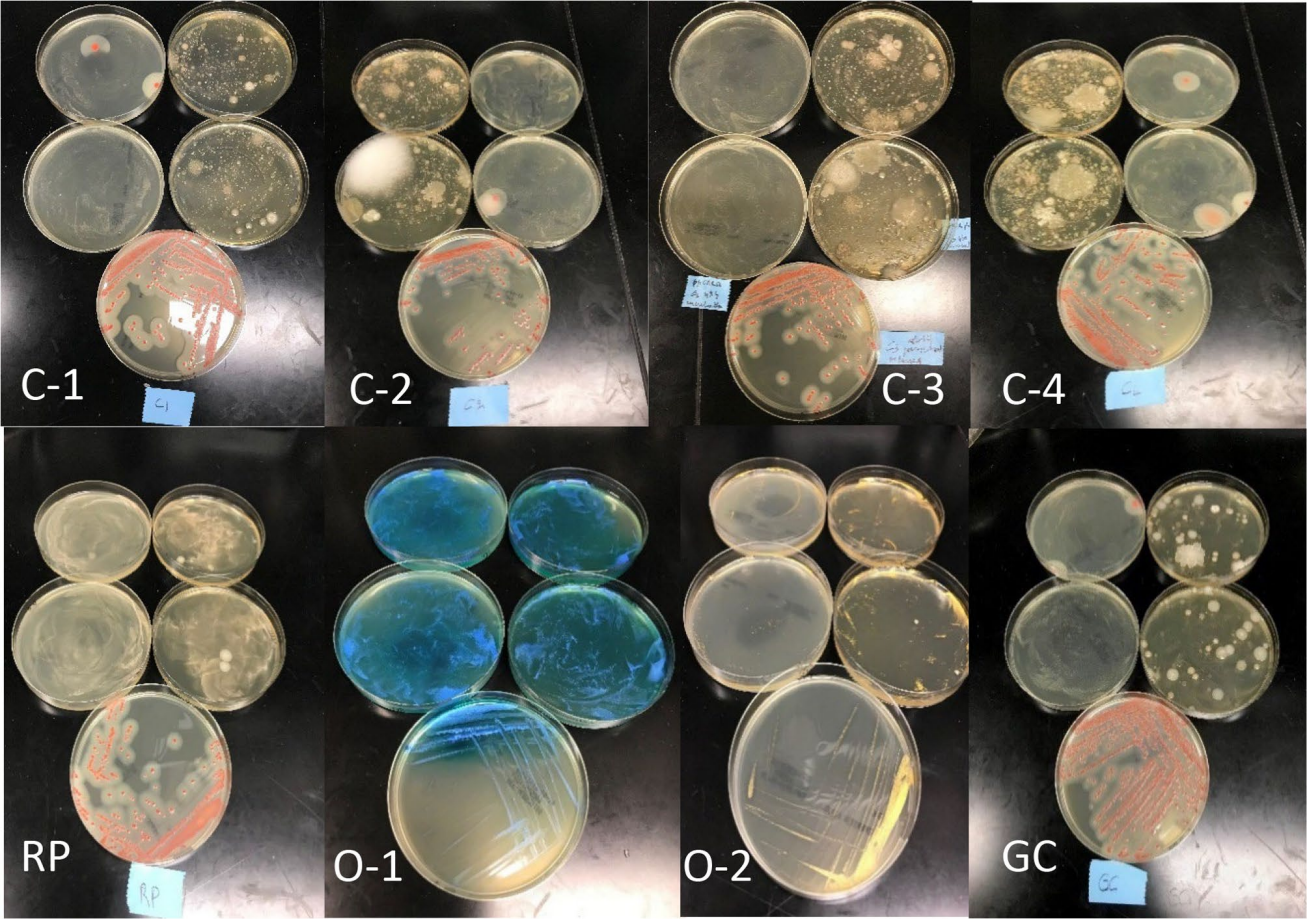
Detection of B. cereus on BACARA plates in powder-type cosmetics. 100 μl of the initial dilution of products (C1, C-2, C-3, C-4, C-5, RP, O-1, 0–2, and GC) spread on MLA and BACARA plates in duplicate (top 4 plates in each image), and a loop streaked on a plate of BACARA after 24 h enrichment (fifth plate at the bottom of each set). (**A**) positive (**B**). cereus result on BACARA shows as orange colonies with a white halo.

After completing both the culture-based method and qPCR analyses of the 8 powder-types cosmetic products, we compared the results acquired by each. Figure 4 shows the culture-based method results.

We found the MLA plates spread with the powder-type samples, which had been previously confirmed as contaminated, grew a heterogeneous population of bacteria from 6 of 8 samples, and 5 of those 6 samples tested positive for the presence of *B. cereus* on BACARA plates. Interestingly, sample C3 only revealed *B. cereus* on BACARA after enrichment, while samples O1 and O2 showed no growth of *B. cereus* (Fig. 4).

Our qPCR assays of the 6 samples which had tested positive according to the culture-based method did show amplification for both 16S rRNA and PLC *B. cereus* targets, giving C_T_ values between 14.96 and 19.46 for the singleplex and 20.29–21.72 for the multiplex assay (Table 6).

**Table 6:** C_T_ values for 16 rRNA and PLC of powder-type products using singleplex and multiplex qPCR and detection on BACARA plates*.

Product	Singleplex		Multiplex		BACARA
	16S rRNA	plc	16S rRNA	plc	
GC	16.6 ± 0.57	20.91 ± na	18.26 ± 1.00	20.85 ± 0.17	p
C-1	17.35 ± 2.01	21.64 ± 1.82	19.04 ± 2.40	21.72 ± 1.87	p
C-2	17.67 ± 1.94	20.62 ± 2.32	19.46 ± 2.70	20.87 ± 2.55	p
C-3	17.11 ± 0.75	21.05 ± 0.72	18.51 ± 1.40	21.19 ± 1.11	p
C-4	16.91 ± 0.57	20.29 ± 0.48	18.30 ± 0.75	20.39 ± 0.82	p
RP	14.96 ± 1.36	20.73 ± 2.03	16.29 ± 2.19	20.61 ± 1.94	p
O-1–1	Und	Und	Und	Und	n
O-2–1	33.46 ± na	38.14 ± na	33.87 ± na	37.90 ± na	n
O-1–2	Und	Und	Und	Und	n
O-2–2	Und	38.72 ± na	Und	Und	n

*Results are shown as C_T_ ± Standard deviation in duplicate for each individual run, 16 S Rdna, *PLC* phosphatidylcholine-specific phospholipase C, *GC* green clay, *C1-4* Pink clay, *RP* Rice powder, *O1-2* Tattoo powder. *P* growth on BACARA plate agar, *n* no growth on BACARA plate agar.

Surprisingly, although Sample O-1 had tested negative according to the culture-based method, our qPCR assays showed amplification of the 16S rRNA (C_T_ value > 33) and PLC (C_T_ value > 37) gene targets in one of our two replicates. However, as these replicates had been pre-enriched and still showed no growth of *B. cereus* when streaked on the BACARA plates, we classified the result as negative.

## Discussion

Current microbiological testing standards for cosmetics in the United States are based on the detection of CFUs, which have not been easily represented by the results of molecular methods such as qPCR. Here we have assessed the utility of qPCR assays developed for *B. cereus* targeting 16S rRNA and PLC targets, in singleplex and multiplex, in pure culture and in cosmetic products. Overall, our qPCR detection assays gave results consistent with those from culture-based methods.

Further, while both singleplex and multiplex assays were successfully applied to naturally and artificially contaminated cosmetic products, using the intercalating PMAxx treatment improved the detection of viable *B. cereus* cells, as opposed to simply detecting targets regardless of cell viability. This advance helps bridge the gap between cultural and molecular detection of pathogens in cosmetics.

To the best of our knowledge, this is the first time that PMAxx has been used as part of a cosmetic microbiological method, and it significantly improved the detection of positive samples. PMAxx dyes efficiently shifted the C_T_ values obtained for 16S rRNA and PLC to higher values, resulting in delta differences of 2.96 and 2.36, respectively, for positive samples, and 7.82 and 7.22, respectively, for negative samples, which is considered as successful^39^. In general, delta C_T_ values were significantly different for the positive and negative samples, and moreover, the effects of PMAxx were strong among the negative samples. C_T_ values of the negative samples were completely shifted towards the end of the cycle or undetermined indicating that PMAxx inhibited the amplification of the DNA from those cells killed by resident preservatives. This was confirmed by the microscopic imaging of the live/dead stained cells from the 14-day aged samples at the High-level of inoculation, which showed a mixture of dead (red) and viable (green) *B. cereus* cells in those samples.

Similar observations have been made in analyses of PMA-treated UHT milks^15^ and in complex microflora ^40^.

Therefore, our qPCR assays, individually or combined, might be used for rapid evaluation of cosmetic raw materials and rapid testing of finished products for the presence of *B. cereus* and will thus reduce the likelihood that products from being contaminated with *B. cereus* reach consumers. Importantly, by using PMAxx with qPCR viable cells can be preferentially detected, removing one of the obstacles to using qPCR assays as rapid tests to support traditional bacterial culture identification in the microbiological safety assessment of cosmetics. The speed of these molecular methods could allow the detection of presumably positive samples, 24 h before the culture-based results.

## Supplementary Information

Supplementary Information.

## Data Availability

All data generated and analyzed during the study are included in this published article. Supplementary raw data supporting the conclusions of this manuscript will be made available by the corresponding authors, without undue reservation, to any qualified researcher.
