# Supplementary material for: qPCR detection of viable Bacillus cereus group cells in cosmetic products
Source: Sci Rep. 2023 Mar 18;13:4477. doi: 10.1038/s41598-023-31128-3 (PMC10024758; doi:10.1038/s41598-023-31128-3)
Supplement: Supplementary file 1 — Supplementary Information. [file 41598_2023_31128_MOESM1_ESM.docx]

**Table:** Strains used to test the inclusivity/exclusivity of the qPCR assays and culture method plating results^a,b^

| Bacterial Species | Strain ID | 16S rRNA | PLC | 16S rRNA/PLC | BACARA  Plates | Origin |
| --- | --- | --- | --- | --- | --- | --- |
| *B. cereus* (112) | 3A | + | + | +/+ | + | Eye shadow FDA (F) |
|  | 3B | + | + | +/+ | + | Eye shadow (F) |
|  | 3C | + | + | +/+ | + | Eye shadow (F) |
|  | 8A | + | + | +/+ | + | Eye shadow (F) |
|  | 8B | + | + | +/+ | + | Eye shadow (F) |
|  | 8C | + | + | +/+ | + | Eye shadow (F) |
|  | so | + | + | +/+ | + | Clay (F) |
|  | Sub A | + | + | +/+ | + | Baby wipes (F) |
|  | Sub B | + | + | +/+ | + | Baby wipes (F) |
|  | Sub C | + | + | +/+ | + | Baby wipes (F) |
|  | Sub D | + | + | +/+ | + | Baby wipes (F) |
|  | Sub E | + | + | +/+ | + | Baby wipes (F) |
|  | Sub F | + | + | +/+ | + | Baby wipes (F) |
|  | Sub G | + | + | +/+ | + | Baby wipes (F) |
|  | Sub H | + | + | +/+ | + | Baby wipes (F) |
|  | Sub I | + | + | +/+ | + | Baby wipes (F) |
|  | Sub J | + | + | +/+ | + | Baby wipes (F) |
|  | Sub K | + | + | +/+ | + | Baby wipes (F) |
|  | Sub L | + | + | +/+ | + | Baby wipes (F) |
|  | Sub M | + | + | +/+ | + | Baby wipes (F) |
|  | Sub N | + | + | +/+ | + | Baby wipes (F) |
|  | Sub O | + | + | +/+ | + | Baby wipes (F) |
|  | Sub P | + | + | +/+ | + | Baby wipes (F) |
|  | Sub Q | + | + | +/+ | + | Baby wipes (F) |
|  | Sub R | + | + | +/+ | + | Baby wipes (F) |
|  | Sub S | + | + | +/+ | + | Baby wipes (F) |
|  | Sub T | + | + | +/+ | + | Baby wipes (F) |
|  | Sub U | + | + | +/+ | + | Baby wipes (F) |
|  | Sub V | + | + | +/+ | + | Baby wipes (F) |
|  | Sub W | + | + | +/+ | + | Baby wipes (F) |
|  | Sub X | + | + | +/+ | + | Baby wipes (F) |
|  | Sub Y | + | + | +/+ | + | Baby wipes (F) |
|  | Sub Z | + | + | +/+ | + | Baby wipes (F) |
|  | Sub AA | + | + | +/+ | + | Baby wipes (F) |
|  | Sub BB | + | + | +/+ | + | Baby wipes (F) |
|  | Sub CC | + | + | +/+ | + | Baby wipes (F) |
|  | Sub DD | + | + | +/+ | + | Baby wipes (F) |
|  | Sub EE | + | + | +/+ | + | Baby wipes (F) |
|  | Sub FF | + | + | +/+ | + | Baby wipes (F) |
|  | Sub GG | + | + | +/+ | + | Baby wipes (F) |
|  | Sub HH | + | + | +/+ | + | Baby wipes (F) |
|  | Sub II | + | + | +/+ | + | Baby wipes (F) |
|  | Sub JJ | + | + | +/+ | + | Baby wipes (F) |
|  | Sub KK | + | + | +/+ | + | Baby wipes (F) |
|  | Sub LL | + | + | +/+ | + | Baby wipes (F) |
|  | Sub MM | + | + | +/+ | + | Baby wipes (F) |
|  | Sub NN | + | + | +/+ | + | Baby wipes (F) |
|  | subO2 | + | + | +/+ | + | Baby wipes (F) |
|  | subP2 | + | + | +/+ | + | Baby wipes (F) |
|  | subQ2 | + | + | +/+ | + | Baby wipes (F) |
|  | subR2 | + | + | +/+ | + | Baby wipes (F) |
|  | subS2 | + | + | +/+ | + | Baby wipes (F) |
|  | subT2 | + | + | +/+ | + | Baby wipes (F) |
|  | subU2 | + | + | +/+ | + | Baby wipes (F) |
|  | subV2 | + | + | +/+ | + | Baby wipes (F) |
|  | subS21.7.16 | + | + | +/+ | + | Baby wipes (F) |
|  | subT21.7.16 | + | + | +/+ | + | Baby wipes (F) |
|  | subU21.7.16 | + | + | +/+ | + | Baby wipes (F) |
|  | subV21.7.16 | + | + | +/+ | + | Baby wipes (F) |
|  | subW21.7.16 | + | + | +/+ | + | Baby wipes (F) |
|  | subX21.7.16 | + | + | +/+ | + | Baby wipes (F) |
|  | 1-A | + | + | +/+ | + | Baby wipes (F) |
|  | 1-B | + | + | +/+ | + | Baby wipes (F) |
|  | 1-C | + | + | +/+ | + | Baby wipes (F) |
|  | 1-D | + | + | +/+ | + | Baby wipes (F) |
|  | 1-E | + | + | +/+ | + | Baby wipes (F) |
|  | 1-F | + | + | +/+ | + | Baby wipes (F) |
|  | 1-G | + | + | +/+ | + | Baby wipes (F) |
|  | 1-H | + | + | +/+ | + | Baby wipes (F) |
|  | 1-I | + | + | +/+ | + | Baby wipes (F) |
|  | 1-J | + | + | +/+ | + | Baby wipes (F) |
|  | 1-K | + | + | +/+ | + | Baby wipes (F) |
|  | 1-L | + | - | +/- | - | Baby wipes (F) |
|  | 2-A | + | + | +/+ | + | Baby wipes (F) |
|  | 2-B | + | + | +/+ | + | Baby wipes (F) |
|  | 2-C | + | + | +/+ | + | Baby wipes (F) |
|  | 2-D | + | + | +/+ | + | Baby wipes (F) |
|  | 2-E | + | + | +/+ | + | Baby wipes (F) |
|  | 4-A | + | + | +/+ | + | Baby wipes (F) |
|  | 4-B | + | + | +/+ | + | Baby wipes (F) |
|  | 4-C | + | + | +/+ | + | Baby wipes (F) |
|  | F3A | + | + | +/+ | + | Food (F) |
|  | F4A | + | + | +/+ | + | Food (F) |
|  | F26 | + | + | +/+ | + | Food (F) |
|  | F96 | + | + | +/+ | + | Food (F) |
|  | FM77 | + | + | +/+ | + | Food (F) |
|  | TJL14 | + | + | +/+ | + | Food (F) |
|  | FTOL-12 | + | + | +/+ | + | Food (F) |
|  | 60006 | + | + | +/+ | + | R. Bennett |
|  | F13061 | + | + | +/+ | + | F |
|  | F180WPB | + | + | +/+ | + | Food (F) |
|  | BAaC | + | + | +/+ | + | F |
|  | 6A6 | + | + | +/+ | + | Commercial probiotic Company S (F) |
|  | 6A7 | + | + | +/+ | + | Commercial probiotic Company Bio (F) |
|  | 6A8 | + | + | +/+ | + | Commercial probiotic Company Bac (F) |
|  | B6A9 | + | + | +/+ | + | F |
|  | 13472 | + | + | +/+ | + | ATCC |
|  | 10987 | + | + | +/+ | + | ATCC |
|  | B6A16 | + | + | +/+ | + | Infant cereal (F) |
|  | 15816 | + | + | +/+ | + | ATCC |
|  | BGG1 | + | + | +/+ | + | gerQA2 mutant (F) |
|  | BGG2 | + | + | +/+ | + | gerlA5 mutant (F) |
|  | BGG3 | + | + | +/+ | + | Ala-1 mutant (F) |
|  | B6E1 | + | + | +/+ | + | F |
|  | 10702 | + | + | +/+ | + | ATCC |
|  | 13472 | + | + | +/+ | + | ATCC |
|  | 14579 | + | + | +/+ | + | ATCC |
|  | 33018 | + | + | +/+ | + | ATCC |
|  | 33019 | + | + | +/+ | + | ATCC |
|  | 49063 | + | + | +/+ | + | ATCC |
|  | 49064 | + | + | +/+ | + | ATCC |
|  | 55055 | + | + | +/+ | + | ATCC |
| *B. thuringiensis* (27) | 5A | + | + | +/+ | + | Eye shadow (F) |
|  | 5B | + | + | +/+ | + | Eye shadow (F) |
|  | 5C | + | + | +/+ | + | Eye shadow (F) |
|  | 7A | + | + | +/+ | + | Eye shadow (F) |
|  | 7B | + | + | +/+ | + | Eye shadow (F) |
|  | 7C | + | + | +/+ | + | Eye shadow (F) |
|  | Abbott | + | + | +/+ | + | F |
|  | Alensis | + | + | +/+ | + | F |
|  | 4AC1 | + | + | +/+ | + | F |
|  | 4AX1 | + | + | +/+ | + | Russia, soil |
|  | 4AY1 | + | + | +/+ | + | Brazil, black pepper |
|  | 4BC1 | + | + | +/+ | + | China, soil |
|  | 4BJ1 | + | + | +/+ | + | Poland, soil |
|  | 4BK1 | + | + | +/+ | + | Spain, soil |
|  | 4BM1 | + | + | +/+ | + | Spain, soil |
|  | 4BX1 | + | + | +/+ | + | China, Sandy soil |
|  | 4BY1 | + | + | +/+ | + | Denmark, scotch pine |
|  | 4BZ1 | + | - | +/- | + | China, black soil |
|  | 125FT8 | + | + | +/+ | + | ATCC |
|  | 1260 | + | + | +/+ | + | ATCC |
|  | 1262 | + | - | +/- | + | ATCC |
|  | 1264 | + | + | +/+ | + | ATCC |
|  | 1266 | + | + | +/+ | + | ATCC |
|  | 1398 | + | + | +/+ | + | ATCC |
|  | 2126 | + | + | +/+ | + | F |
|  | 6458X | + | + | +/+ | + | F |
|  | 35866 | + | + | +/+ | + | ATCC |
| *B. mycoides* (4) | 6A11 | + | + | +/+ | + | From non-potable water tank in vicinity of livestock and abattoir (F) |
|  | 6A12 | + | + | +/+ | + | From oïl sample, UK |
|  | 6A14 | + | + | +/+ | + | From dust sample, UK, in 1979 |
|  | 6462 | + | + | +/+ | + | ATCC |
| Non-*cereus* (38) |  |  |  |  |  |  |
| *B. subtilis* | 2A8T | - | - | -/- | - | Tunisia, desert |
|  | 3A17 | - | - | -/- | - | Mediteranean Sea |
|  | 3A18 | - | - | -/- | - | Soil |
|  | 3A19 | - | - | -/- | - | Soil |
|  | 3A23 | - | - | -/- | - | Infant cereal |
|  | 3A24 | - | - | -/- | - | Trifolium repens rhizosphere |
|  | B14197 | - | - | -/- | - | F |
|  | B14198 | - | - | -/- | - | F |
|  | B14199 | - | - | -/- | - | F |
|  | B14201 | - | - | -/- | - | F |
|  | B14208 | - | - | -/- | - | F |
|  | B14223 | - | - | -/- | - | F |
|  | B14202 | - | - | -/- | - | F |
|  | B14203 | - | - | -/- | - | F |
|  | B14204 | - | - | -/- | - | F |
|  | 6051 | - | - | -/- | - | ATCC |
|  | 15563 | - | - | -/- | - | ATCC |
| *B. pumilus* | 8A4 | - | + | -/+ | - | Mediterranean Sea (F) |
|  | 7061 | - | - | -/- | - | ATCC |
| *B. licheniformis* | 12759 | - | - | -/- | - | F |
|  | 21308 | - | - | -/- | - | F |
|  | B14224 | - | - | -/- | - | F |
|  | B14225 | - | - | -/- | - | F |
|  | B14227 | - | - | -/- | - | F |
|  | 14580 | - | - | -/- | - | F |
| *B. megaterium* | 11561 | - | - | -/- | - | F |
|  | NY | - | - | -/- | - | Mask, FDA |
| *B. mojavensis* | 28A1T | - | - | -/- | - | Mojave desert (F) |
|  | 28A2 | - | - | -/- | - | Mojave desert (F) |
|  | 28A3 | - | - | -/- | - | Mojave desert (F) |
| *B. carboniphilus* | 100AIT | - | - | -/- | - | From the air on plates Antibiotic Medium No. 3 agar containing carbon graphite |
| *B. clausii* | 15A3 | - | - | -/- | - | Commercial probiotic, Company Sa |
|  | 17A1 | - | - | -/- | - | Commercial probiotic, Company D |
| *B. coagulans* | 61A1T | - | - | -/- | - | From evaporated milk (F) |
| *Brevibacillus* | 21991 | - | - | -/- | - | F |
| *Paenibacillus alvei* | 33A1 | - | - | -/- | - | Dead mosquito larvae (F) |
|  | 33A2 | - | - | -/- | - | Dead mosquito larvae (F) |
|  | 33A3 | - | - | -/- | - | Dead mosquito larvae (F) |
| Non-*Bacillus* spp. (31) |  |  |  |  |  |  |
| *-Burkholderia -cepacia* | 53267 | - | - | -/- | - | ATCC |
| *-Candida albicans* | 20308 | - | - | -/- | + | ATCC |
| *E. coli* | O103:H25 | - | - | -/- | - | F 0752; Human (F) |
|  | O104:H21 | - | - | -/- | - | F 0898 |
|  | O104 | - | - | -/- | - | German Isolate; F0631 |
|  | O111:H11 | - | - | -/- | - | Cow; F 0877 |
|  | O113:H21 | - | - | -/- | - | Australia, clinical; F0716 |
|  | O121 | - | - | -/- | - | F 0806 |
|  | O145 | - | - | -/- | - | F 0812 |
|  | O157:H7 | - | - | -/- | - | F 0787; Ohio Department of Agriculture |
|  | O26:H11 | - | - | -/- | - | Cow ; F 0757 |
|  | O45 | - | - | -/- | - | MSU Isolates ; F 0818 |
|  | MG1655 | - | - | -/- | - | Dr. Rachel Binet |
| *L. innocua* | 1453 | - | - | -/- | - | French brie (F) |
|  | 2492 | - | - | -/- | - | F |
| *L. ivanovii* | 1454 | - | - | -/- | - | French brie (F) |
|  | 0140 | - | - | -/- | - | F |
| *L. monocytogenes* | 2464 | - | - | -/- | - | Intact peach (F) |
|  | 2465 | - | - | -/- | - | Caramel apple (F) |
| *P. aeruginosa* | 866 | - | - | -/- | - | Beef/Meat (F) |
|  | 0014 | - | - | -/- | - | ATCC |
| *P. fluroputida* | 1582 | - | - | -/- | - | F |
| *P.* spp. | 2820 | - | - | -/- | - | ATCC ; |
| *S.* Enterica | 0044 | - | - | -/- | - | Food, fish (F) |
| *S. aureus* | 485 / 86 | - | - | -/- | + | R. Bennett |
|  | 21293 | - | - | -/- | + | ATCC |
|  | 49969 / 654 | - | - | -/- | + | Wound (F) |
|  | 54616 / 655 | - | - | -/- | + | Wound (F) |
|  | 2821 | - | - | -/- | + | ATCC |
| *S. intermedius* | 87-312142 | - | - | -/- | + | Dog gingiva (F) |
|  | 87-347143 | - | - | -/- | + | Dog gingiva (F) |

ATCC: American Type Collection Center; F: U.S. FDA Stock Collection; gerQA2: inosine related-germinant operon; gerIA5: alanine related germinant operon; Ala-1: alanine. a represents qPCR results and b represents results on BACARA plates.
